# S208. PREDICTORS OF RESPONSE TO COGNITIVE REMEDIATION THERAPY: SYSTEMATIC REVIEW OF LITERATURE

**DOI:** 10.1093/schbul/sby018.995

**Published:** 2018-04-01

**Authors:** Maree Reser, Reneta Slikboer, Susan Rossell

**Affiliations:** Swinburne University of Technology

## Abstract

**Background:**

Impaired cognitive functioning is considered a core aspect of schizophrenia and is associated with poorer functional outcomes. Proving only marginally responsive to pharmacological interventions, there has been an acceleration of research investigating the efficacy of cognitive remediation therapy (CRT) in ameliorating cognitive deficits. While small to moderate effect sizes have been reported, closer examination suggests 40–60% of participants fail to realise a benefit. To improve both efficacy and effectiveness, better understanding of the factors that predict cognitive response to CRT is needed. To date, no systematic review of the evidence base has been conducted. We aimed to address that gap by providing a synthesis of predictor variables, whether they were moderators, mediators or predictors, of cognitive response to CRT.

**Methods:**

An electronic database search was conducted across Scopus, Web of Science and PsychINFO databases and the Cochrane Collaboration Controlled Trials Register for all years until 30/09/2017. Reference lists of published meta-analyses and review articles were hand searched. Eligibility assessment was performed independently in an unblinded standardised manner by two reviewers. Studies that included a CRT arm, had a majority (≥70%) schizophrenia / schizoaffective disorder participants, had at least one training-distinct pre-post measure of cognition and at least one predictor of cognitive outcome were included. Studies that incorporated social cognitive training and/or adjunctive rehabilitation were excluded. A box-score analysis of predictor variables was conducted and the quality of the predictor evidence was assessed.

**Results:**

Of 417 records extracted, 37 articles considering 1,499 overlapping CRT participants (2,423 full sample) were included in the final synthesis. On average, participants were in their mid-thirties, majority male, with approximately 12 years education. CRT trial arm size averaged 41.64 participants. Overall, 72 distinct predictors of cognitive response were identified, with an average 4.89 predictor variables considered across an average 3.95 cognitive domains per article. Of these, 42 were analysed once and 10 twice. Discussion focused on the 20 predictors examined a minimum 3 times. Highlighting the exploratory nature of predictor research to-date, few studies were theory driven or evidence based and fewer still were undertaken with a priori hypotheses. Only a handful of analyses included tests of interaction.

**Discussion:**

Few of the currently examined predictors of cognitive response to CRT are significant when examined as a systematic review. The influence of age was the most frequently examined predictor, with a majority of articles finding no association. The strongest category level trend was found in baseline cognition, especially in reasoning and problem solving and working memory domains, which was more strongly predictive of within domain improvement. Training task improvement or “learning potential” was the most notable cross-domain predictor of cognitive outcome, though this was limited to three articles and warrants further investigation. It remains unclear why up to 60% of participants do not receive benefit from CRT. There is a need to look beyond the usual candidates, to pool data both to support methodologically robust, large-scale investigations and to better account for cross-population variability.

